# Genetic Counseling for an Infertile Couple With Premature Chromatid Separation (PCS) Syndrome: A Case Report

**DOI:** 10.7759/cureus.56921

**Published:** 2024-03-25

**Authors:** Sagiri Taguchi, Terumi Hayashi, Noriko Watanabe, Yoshihiro Tada, Takashi Matsubara, Giannina Calongos, Kiko Yamamoto, Risa Fujishima, Sayaka Imoto, Miyako Funabiki, Yoshitaka Nakamura

**Affiliations:** 1 In Vitro Fertilization Center, Oak Clinic, Osaka, JPN

**Keywords:** case report, live birth, premature chromatid separation (pcs) syndrome, infertile couple, genetic counseling

## Abstract

We report the first case of successful genetic counseling for an infertile couple with premature chromatid separation (PCS) syndrome. After our careful genetic counseling, the couple decided to continue infertility treatment. As a result, they gave birth to a baby (girl: 2,930 g) by caesarean section in May 2018. To our knowledge, there have not been any published reports regarding genetic counseling for an infertile couple with PCS after PubMed, EMBASE, and Web of Science searches until March 2024.

## Introduction

Premature chromatid separation (PCS) is a rare genetic syndrome [1-4]. Infertile couples with PCS may have negative impressions or experience bad outcomes. However, after PubMed, EMBASE, and Web of Science searches until March 2024, to our knowledge, there have not been any published reports regarding genetic counseling for an infertile couple with PCS. According to the guidelines of the Japanese Association of Medical Sciences, genetic counseling provides not only information but also psychological and social support so that the patient/examinee can autonomously make a decision [5]. However, a case report was not shown about genetic counseling for an infertile couple with PCS [3]. Therefore, the objective of our case report is to show the clinical benefit of genetic counseling for an infertile couple with PCS syndrome, according to the Japanese Medical Society [5]. 

## Case presentation

**Figure 1:**
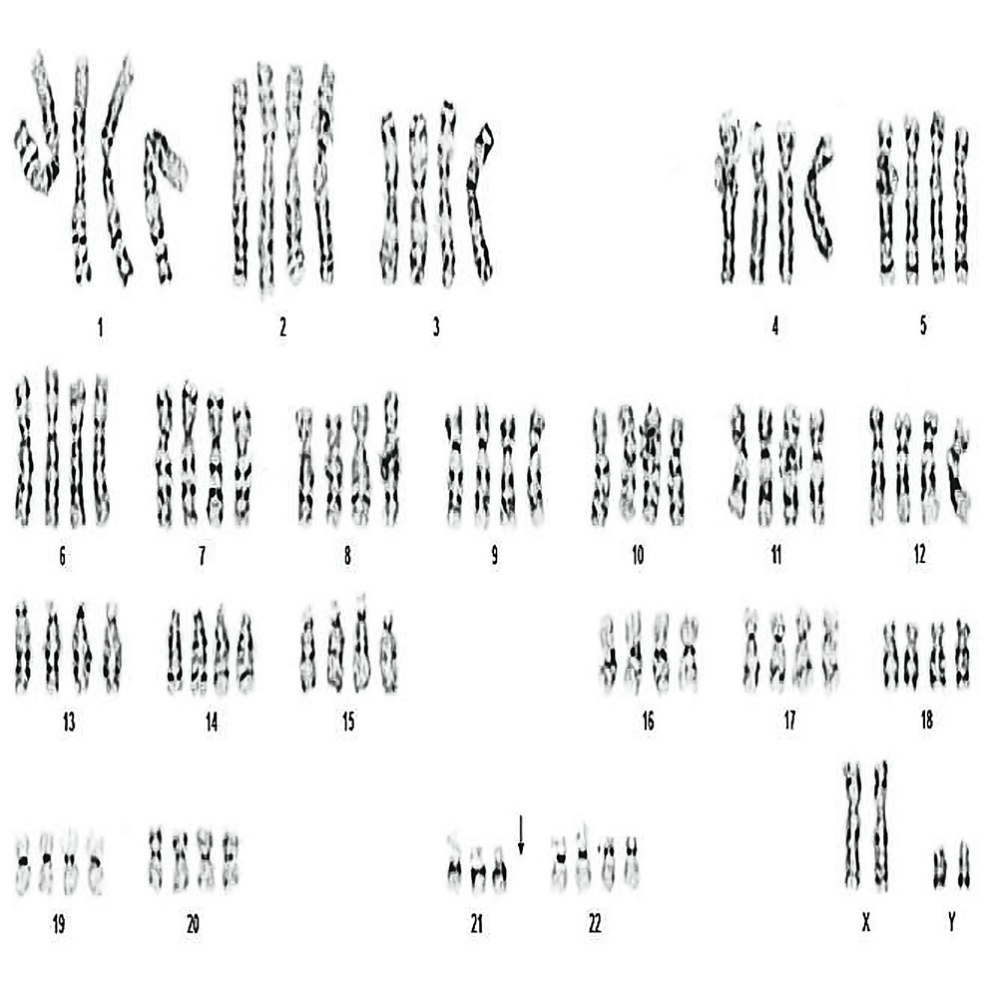
An abnormal karyotype of the fetus (91, XXYY, -21).

In this case, the wife, aged 33, experienced her third miscarriage at 10 weeks and two days of gestation. The couple visited us for genetic counseling and tests. The results of genetic testing, conducted in August 2016, showed an abnormal karyotype of the fetus (91, XXYY, -21) (Figure 1). 

**Figure 2:**
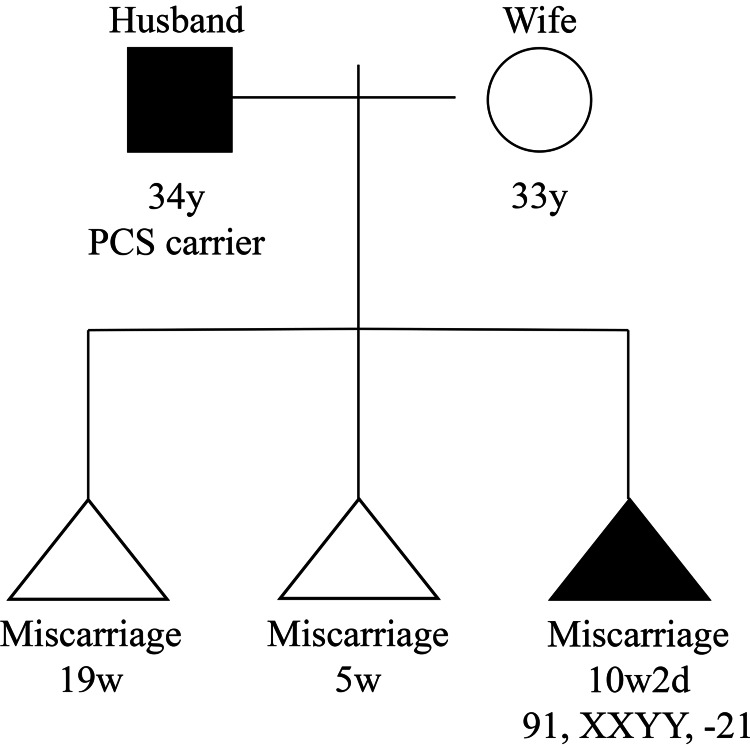
A family tree. PCS carrier should be read as a "PCS-asymptomatic." PCS: premature chromatid separation.

Furthermore, her husband (34 years old) was a patient with PCS-asymptomatic in October 2016. A family tree indicating their situation is shown in Figure 2.

**Figure 3:**
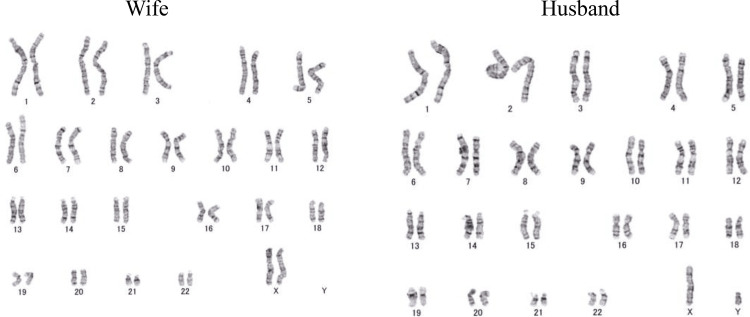
Wife’s and husband’s results for chromosome tests.

We explained to the couple, who wanted to understand the cause of the miscarriage, as follows. After chromosome testing, the fetal karyotype was found to be 91, XXYY, -21 (Figure 1). Furthermore, the karyotype (Figure 1) was from their third pregnancy (Figure 2). It was highly possible that this chromosomal abnormality was the cause of the miscarriage. After chromosome tests, we explained to them that the wife’s results indicate a normal karyotype and the husband’s results indicate 46,XY, also a normal karyotype (Figure 3).

However, the G-band test results of the husband showed PCS (27 out of 200 cells in peripheral blood). Therefore, it can be determined that the husband is PCS-asymptomatic.

Furthermore, we carefully explained to them the basics of PCS syndrome as follows. PCS follows an autosomal dominant inheritance pattern, but individuals with PCS do not usually have symptoms. It typically presents as a cytogenetic alteration with no associated phenotype, as seen in this case. While we categorically affirm that PCS is not associated with the infertility in this couple, there are anecdotal reports of a family with subfertility and PCS [3,4]. As there is a lack of research to establish fertility in individuals with PCS, further biological studies will be needed.

In response to their concerns about their future child, we carefully explained that it will be possible to perform amniocentesis during the wife's pregnancy to ease any concerns they may have at first, and secondly, in vitro fertilization (IVF) cannot prevent PCS from being passed on. Furthermore, as for our IVF protocols (ovarian stimulation protocol, embryo development, and transfer methodology), we used the same method as outlined in the study by Imoto et al. [6]. Moreover, we also recommended that the husband avoid using drugs that act on the polymerization of spindles, such as vincristine, vinblastine, and paclitaxel, because carriers of PCS may be highly sensitive to these drugs. In this way, we were able to give them some useful advice.

As a result of our careful genetic counseling, the couple decided to continue infertility treatment. They successfully gave birth to a baby girl weighing 2,930 g via caesarean section in May 2018. 

## Discussion

In this case, the couple struggled with infertility and experienced miscarriages in the past. Discovering a problem with the husband's chromosomes compounded their upset, as they came to believe that this was the cause of their trouble. Many couples do not fully understand the result of genetic testing, and they experience anxiety, worry, and irritation that could easily be alleviated with discussion and counseling focused on their goals rather than solely focusing on the results, as suggested by Pastore et al. [7] when considering the psychosocial impact of genetic testing.

In this case, the couple was counseled that the new information about PCS status did not indicate any changes to their fertility plans. After our careful genetic counseling, the couple decided to continue with IVF, and they could get a baby.

Currently, research, including previous studies/case reports, is insufficient to establish fertility in individuals with PCS [1-4]. Research have not shown genetic counseling for an infertile couple with PCS syndrome, according to the Japanese Medical Society [1-5]. 

Finally, although the incidental finding of PCS would be useful for the husband's overall health management in our case, the key learning point in this case is to provide useful insights to others in the management of patients with PCS in the context of infertility investigation.

## Conclusions

In conclusion, we would like to emphasize the importance of careful genetic counseling and genetic tests for an infertile couple with PCS in order to avoid patient stress. Our research will be a good step in the management of such patients. Furthermore, in light of educational merits, our study is a first report regarding genetic counseling for an infertile couple with PCS, after PubMed, EMBASE, and Web of Science searches until March 2024.
